# Exploring rice tolerance to salinity and drought stresses through *Piriformospora indica* inoculation: understanding physiological and metabolic adaptations

**DOI:** 10.3389/fpls.2024.1428631

**Published:** 2024-09-24

**Authors:** Ali Raeisi Vanani, Fatemeh Sheikhi Shahrivar, Amin Nouri, Mozhgan Sepehri

**Affiliations:** ^1^ Department of Soil Science, College of Agriculture, Isfahan University of Technology, Isfahan, Iran; ^2^ Department of Plant and Soil Science, College of Agriculture, Food, and Environment, University of Kentucky, Lexington, KY, United States; ^3^ Department of Research and Development, Monty’s Plant Food, Louisville, KY, United States; ^4^ Department of Soil Science, College of Agriculture, Shiraz University, Shiraz, Iran

**Keywords:** Piriformospora indica, inoculation, fungi, salinity stress, drought stress, rice, catalase enzyme, nutrients

## Abstract

Drought and salinity are significant challenges to global food security. This study investigated the interactive impacts of *Piriformospora indica* inoculation with salinity and drought stresses on rice. Two greenhouse experiments were conducted. The first experiment evaluated two *P. indica* inoculation levels and three salinity levels (0-, 50-, and 100-mM sodium chloride), while the subsequent experiment assessed two inoculation levels under three drought intensities (25%, 50%, and 100% of available water content). *P. indica* spores were inoculated following optimized seed disinfection and germination processes. The shoot and root biomass under salinity stress were consistently higher in inoculated plants compared to controls. Sodium concentrations in shoots and roots exhibited an overall upward trend, with the trend being less pronounced in inoculated plants due to increased potassium uptake. Under salinity stress, nitrogen, magnesium, and calcium concentrations significantly increased in inoculated plants. With increasing salinity, there was a significant increase in catalase enzyme activity and soluble carbohydrate concentrations across all treatments, with a greater increase in inoculated plants. Plants under drought stress experienced reduced root and shoot biomass, but inoculated plants maintained higher biomass. Increasing drought stress led to decreased nitrogen, magnesium, and calcium concentrations in all treatments, with the reduction being less severe in inoculated plants. Catalase enzyme activity and carbohydrate increased with rising drought stress, with the increase being more pronounced in inoculated plants compared to non-inoculated ones. By promoting plant growth, nutrient uptake, and stress tolerance, *P. indica* inoculation has a significant potential to enhance crop productivity in extreme climate conditions.

## 1 Introduction

Drought and salinity are key environmental stressors that significantly hinder crop production worldwide, with a major impact on crop yield (El Sabagh et al., 2020). The global extent of salt affected soils is estimated to range between 8.3 and 11.7 Mkm^2^ (Hassani et al., 2021). This corresponds approximately 16.6 to 23.4% of the total global arable land area (~50 Mkm^2^) (FAO, 2021), although precise data remains unavailable. A considerable proportion of salt-affected soils is concentrated in arid and semi-arid climatic zones in Asia and Africa with precipitation-to evaporation ratios typically around 0.75 or less (Brady et al., 2008). Dryland agriculture is globally challenged by progressive water scarcity. The issue is further compounded by soil salinity, amplifying the detrimental effects of drought on soil productivity. The salinity resembles drought conditions by reducing the osmotic potential of the soil solution, thereby impeding the plant’s ability to uptake water, even when it is present in the root zone (Stavi et al., 2021). Soil salinity may arise from natural processes, such as precipitation, aeolian deposits, weathering of parent material or surface and brackish groundwater flow. The secondary origin of soluble salts is predominantly attributed to saline irrigation water and fertilizers (Rezapour et al., 2023). The concurrent influence of drought and salinity poses a substantial threat to dryland crop production, leading to various economic, environmental, and social challenges. Future projection indicates a substantial rise, surpassing a quintuple increment, in water deficit within arid and semi-arid climatic regions, consequent to a 3°C elevation in global temperature by the end of 21^st^ century (Dai, 2011). Maintaining ample water in the root zone is key to increasing the osmotic potential in saline soil solution, thereby facilitating water and nutrient uptake by plant roots. Consequently, the detrimental effect of salinity on crop growth will be likely further accentuated under the anticipated drier future (Bartlett et al., 2012). Drought-salinity impact leads to reduced photosynthesis, stomatal conductance, plant vigor, and root growth, thereby reducing crop yield. Among agricultural crops, rice is particularly susceptible to the deleterious impacts of abiotic stresses such as drought and salinity. Rice accounts for 50-80% of the calorie intake for nearly three billion individuals worldwide and is recognized as the second most significant global commodity crop (Ghosh et al., 2016). In response to the imposition of low external water potential, plants invoke a defensive strategy by increasing leaf area and shoot growth. However, this defensive mechanism often results in a diversion of substantial plant energy toward vegetative rather than productive growth, thereby contributing substantially to crop failure. Recent studies have linked drought induced changes in respiration rates, metabolism, and cell wall properties to the severity of drought stress (Seleiman et al., 2021). Moreover, drought-salinity tension can lead to a reduction in amino acid content and causing the toxicity of certain ions such as boron, chloride, and sodium within plant tissues, adversely affecting plants’ physiological functions and food quality (Ma et al., 2020; Shahid et al., 2020; Seleiman et al., 2021). The leaching of soluble salts through surface irrigation is the prevalent remediation approach for saline-sodic soils. Nevertheless, initially limited water resources in semi-arid agriculture and compromised quality of leaching outflow impel growers and policymakers to explore more resource-efficient strategies. The remediation of concomitant salinity-drought issues presents a complex challenge owing to the highly variable intensity and continual degradation of salt-affected soils. Among available strategies, the adoption of bioremediation approaches such as the inoculation of plant with beneficial soil microorganisms like mycorrhizal fungi and endophytes, has been shown to enhance plant resilience to environmental stressors (Hanaka et al., 2021). Endophytic bacteria are the plant beneficial bacteria which by creating genetic, physiological, and ecological changes in their host plants, increase the plant development possibility and root’s efficiency per unit area in salinity and drought conditions. *Piriformospora indica (P. indica)*, Trichoderma species *(Trichoderma* spp), Bacillus species (*Bacillus* spp), and Arbuscular Mycorrhizal Fungi (AMF) are examples of endophytes known to play a positive role in improving stress tolerance in plants (Verma et al., 1999; Contreras-Cornejo et al., 2014).

*P. indica* is one of the endophytic fungi that can coexist with many agricultural plants and is also able to grow in artificial culture environments. *P. indica* is known to be effective in controlling fungal pathogens with a great potential to increase the growth of plants and reduce stress-causing conditions (Yaghoubian et al., 2022). Inoculation with *P. indica* has been shown to have a positive impact on the physiological and metabolic characteristics of rice plants under salinity and drought stress. Studies have demonstrated that inoculation with *P. indica* increases photosynthesis, root growth, and nutrient uptake, as well as decreasing water use, thus improving the plant’s tolerance to drought and salinity stresses (Yaghoubian et al., 2014). In addition, inoculation with *P. indica* increases the production of secondary metabolites which are beneficial to the plant’s physiological processes, such as antioxidants and anti-inflammatories. *P. indica* orchestrates a comprehensive defense strategy, inducing stress responsive genes, modulating reactive oxygen species, and enhancing abscisic acid signaling to help plants cope with salinity and drought stress. Its biocontrol properties confer systemic resistance, protecting plants from pathogens and enhancing overall stress resistance (Smith and Williams, 2024).

In light of the intensified salinity and drought stresses impacting key agricultural commodities, especially in arid and semi-arid regions, understanding the efficacy of remedial strategies in sustaining plant nutrition and vigor under stress is essential for food security (Yoder et al., 2022). Despite the demonstrated efficacy of plant inoculation with endophytes in enhancing plant tolerance to salinity and drought stresses, there remains a scarcity of detailed understanding the complete spectrum of essential plant nutrients, biochemical indicators, and morphological features affected by endophyte inoculation under salinity and drought stresses. This study aims to address this gap by investigating the impacts of salinity and drought, with a specific focus on *P. indica*, employing different growth conditions, and providing a comprehensive quantitative assessment of growth indices and physiological responses. The insights garnered from this research contribute valuable nuances to the current understanding of how specific endophytes can bolster crop resilience amidst complex environmental stresses.

## 2 Materials and methods

### 2.1 Treatments and study design

Two greenhouse experiments were conducted, employing a Completely Randomized Design (CRD) with a factorial layout and 4 replicates. In the initial experiment, two levels of *Piriformospora indica*—specifically, inoculated, and non-inoculated seeds—each were investigated in conjunction with three levels of salinity: 0-, 50-, and 100-mM sodium chloride solutions. The subsequent experiment followed a parallel design, incorporating two levels of *P. indica* inoculated and non-inoculated treatments. Notably, the second experiment diverged by examining a factor encompassing three drought levels, corresponding to 25%, 50%, and 100% of available water content (AWC). A complex culture medium was prepared (**Table 1**) for propagation and inoculation of *Piriformospora indica*. *P. indica* cultures were incubated at a controlled temperature of 24°C to ensure optimal spore production (Verma et al., 1999). Cultured *P. indica* were transferred to laminar hood. The sterile rubber and water-twin (20 L) were used for collecting spores from the surface of the medium. Once the liquid containing spores was filtered through paper, it was transferred into multiple 50 ml falcon tubes. These tubes underwent a 4minute vortexing and a 2-minute sonication, followed by centrifugation at 7°C and 500 rotations per minute. After centrifugation, the supernatant was discarded, water-twin was also added to the residual sediment, then sonicid was applied again and the processes were repeated three times to ensure the effective isolation and purification of *P. indica* spores.

**Table 1:** The formula of *P. indica* culture medium (per 1 liter).

Glucose	Fungi Yeast	Casamino Acid	Salt solution	Peptone	Microelement	Agar
20 g	1 g	1 g	50 ml	2 g	1 ml	15 g

### 2.2 Seed inoculation for salinity and drought stresses

Hashemi rice (*Oryza sativa L*.) underwent a disinfection process involving exposure to 95% ethanol for 30 seconds, followed by immersion in a 1% sodium hypochlorite solution for 5 minutes. To mitigate potential toxic effects from sodium hypochlorite, the sterilized seeds were rinsed five times with sterile distilled water. For germination, the seeds were arranged on sterile filter paper in a petri dish at appropriate intervals and subsequently incubated at a controlled temperature of 20°C. To prepare seeds for salinity and drought stresses, the seedling trays were filled with sterile sand, and seeds of uniform size were selected. These seeds were subjected to a 3-hour agitation at 80 rpm in a 2% Tween water solution containing 5×10^6^ spores, ensuring complete spore coverage. Subsequently, the seeds were placed in sterilized sand trays, with 1 ml of the *P. indica* inoculant solution added to each seed. To ascertain successful symbiosis between *P. indica* and the host plant, the inoculated seeds were transitioned to a hydroponic culture medium after a 72-hour incubation period.

### 2.3 Plant cultivation under salinity and drought stresses

**Figure 1:**
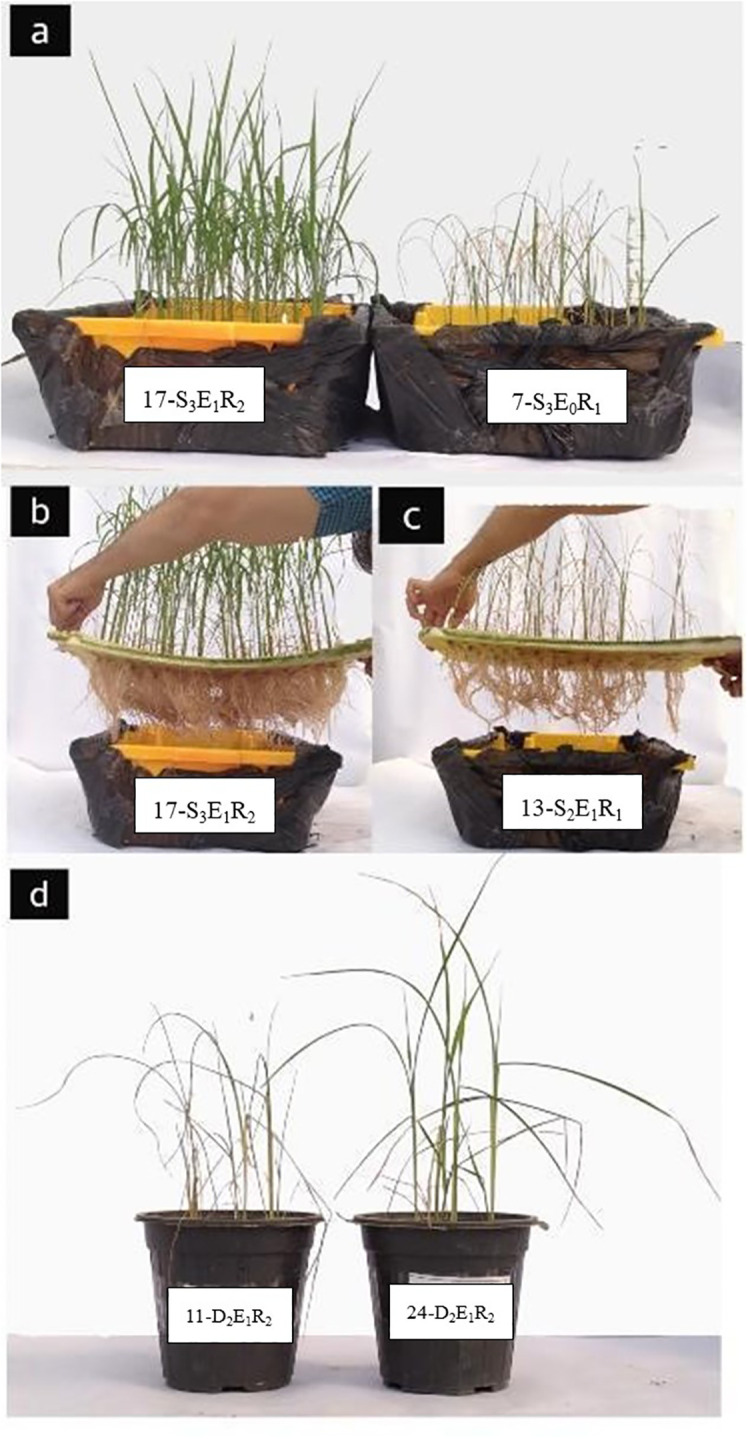
Visual representation of the experimental setup and comparisons of treatments. **(A)** Example demonstration of shoot biomass comparison between inoculated (left) and non-inoculated (right) rice crop at a salinity level of 100 mM. Corresponding root biomass comparison of inoculated **(B)** versus non-inoculated **(C)** rice crop. **(D)** Visual contrast in shoot biomass between inoculated (right) and non-inoculated (left) crop under 50% field capacity (FC) drought stress. The letters D, E, S, and R on the labels denote drought level, presence (E_1_) or absence (E_0_) of inoculation, salinity level, and replicate, respectively.

Four rice seedlings, both inoculated and non-inoculated, with comparable sprout sizes, were cultivated in each pot with standardized autoclaved soil (30 minutes at 121°C). Then, the pots were relocated to a greenhouse under a 16-hour light period, daily temperatures ranging from 20 to 30°C, nightly minimum temperatures of 18°C, a relative humidity of 60%, and a light intensity of 10,000 lux, sustained over an 8-week growth period. Distilled water was used daily for irrigation. To induce salinity stress, hydroponic cultivation was implemented. Rice seedlings, ensuring uniform size for both inoculated and non-inoculated samples, were transplanted into hydroponic culture enriched with a nutrient solution. Following transplantation, the seedlings were relocated to the greenhouse and nourished with the Yoshida nutrient solution throughout the 8-week growth period. After about two weeks post-planting, salinity was applied. The salinity gradient comprised three levels (0, 50, and 100 mM sodium chloride), gradually infused into the nutrient solution across three stages to mitigate abrupt stress imposition. The field capacity (FC) and permanent wilting point (PWP) moisture contents were determined employing pressure plate extractors, yielding values of 28.18% and 16.2% by volume, respectively. Considering the constant weight of empty pots and dry soil, the requisite water quantity for stress levels corresponding to 100%, 50%, and 25% of AWC was calculated. The pots were maintained at the FC level for 21 days, extending until the four or five leaves stage, after which the drought stress treatments commenced 21 days post-planting. To maintain the designated moisture levels in each pot, a daily weighing was performed, and water was added. Five weeks after applying the considered stresses and after the end of the plant growth phase and observing the apparent difference in the growth of plants inoculated with *P. indica* under different stress conditions, the plants were excised from the collar, and the roots from both the hydroponic culture and pots were separated. Plant shoot and root were immediately transferred to liquid nitrogen and kept at -80 C to be used for metabolomics experiments (**Figure 1**).

### 2.4 Plant tissue analysis

The plant shoots and roots underwent desiccation in an oven at 70 C for two days. Following cooling, their mass was determined. To measure nutrient content, roots and shoots were rinsed with distilled water, followed by grinding and incineration at 550 C for 2 hours in an oven. Plant ashes were then extracted with 10 ml of 2N hydrochloric acid (HCl) (Lim and Jackson, 1983). Subsequent elemental analysis included the determination of K^+^ and Na^+^ concentrations via atomic absorption spectrophotometry (Perkin-Elmer 5100 PC), N concentration via the Kjeldahl method, and Ca^2+^ and Fe^3+^ concentrations utilizing the atomic absorption device model Perkin Elmer 3030 (Walkley and Black, 1934). Catalase enzyme activity was quantified utilizing a modified methodology based on the reduction of hydrogen peroxide (Bergmeyer, 2012). The spectrophotometer was set at a wavelength of 240 nm. A solution comprising 3 mL of sodium phosphate buffer, 51.4 mL of hydrogen peroxide, and 50 microliters of the extract was prepared and thoroughly mixed before spectrophotometric analysis. Enzyme activity was quantified as μmol min^-1^g^-1^. The total soluble carbohydrates were extracted from fresh tissue and measured using a spectrophotometer at a wavelength of 620 nm. To 0.1 g of dry matter, 10 mL of 70% ethanol was added, and the mixture was refrigerated for one week. After one week, 0.5 mL of the supernatant was taken, and the volume was adjusted to 2 mL with distilled water. To each prepared solution, 1 mL of 5% phenol was added, followed by 5 mL of concentrated sulfuric acid after thorough mixing. A yellow solution was formed, which gradually changed color. This solution was left at room temperature for 30 minutes to cool down and develop the final color. The intensity of the resulting color was then measured using a spectrophotometer at a wavelength of 485 nm.

### 2.5 Statistical analysis

MIXED ANOVA procedure was conducted in SAS (SAS, 2013). Least square means were separated by Fisher’s least significant difference test (LSD) at p < 0.05 significance level. Two levels of *Piriformospora indica* (inoculated and non-inoculated—were considered fixed-effect and salinity and drought levels were considered as factor. Hierarchical cluster analysis employing the Euclidean distance metric and Ward’s minimum variance linkage method was conducted to delineate hierarchical groupings among similar treatments and to assess the consistency of variables across replicates. The analysis was conducted using SciPy library in Python version 3.11.8 in Jupyter Lab environment.

## 3 Results

### 3.1 Effect of inoculation-salinity and inoculation-drought on dry biomass

**Figure 2:**
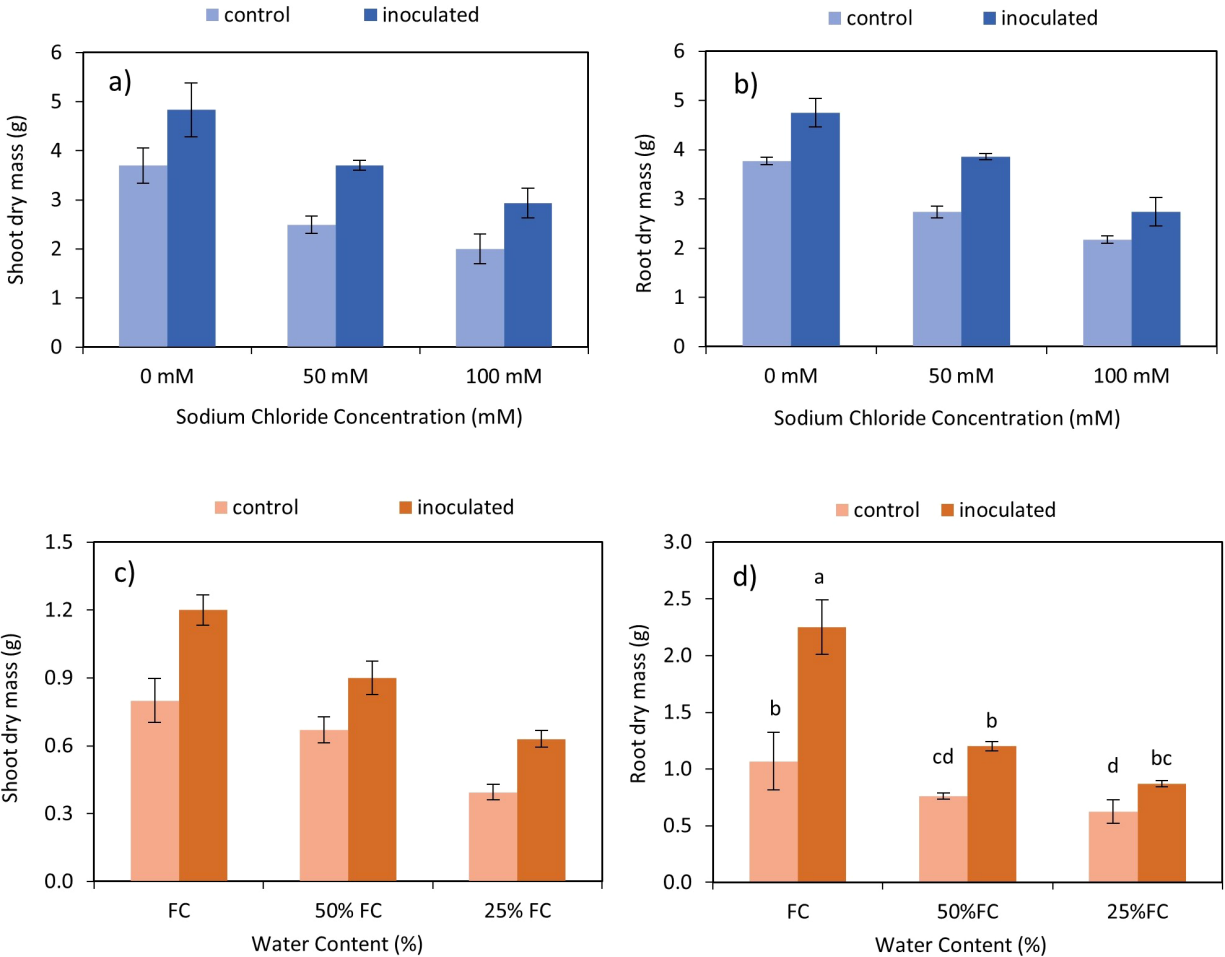
Interactive effect of *P. indica*-salinity on dry mass of shoots **(A)** and roots **(B)** and the interactive effect of *P. indica*-drought on dry mass of shoots **(C)** and roots **(D)**. The letter annotations indicate significant difference among treatments across stress levels according to Fisher’s LSD (p < 0.05). The vertical error bars in the figures denotes standard error.

The results demonstrated statistically significant interactions between *P. indica* and salinity (**Table 2**), as well as *P. indica* and drought (**Table 3**), affecting the dry mass of both shoots and roots. Inoculation with *P. indica* led to an increase in shoot and root dry mass compared to the control across all salinity levels (**Figures 2A, B**). The highest and lowest shoots and roots dry mass was observed in plants inoculated with *P. indica* without salinity stress and non-inoculated plants with 100 mM sodium chloride salinity stress, respectively. The shoot’s dry mass inoculated with *P. indica* was about 1.3 times higher than the shoot’s dry mass in control plants (**Figure 2A**). The root dry mass in inoculated plants under 50- and 100-mM salinity stress was 1.41 g and 1.26 g, respectively. The shoot’s growth under 50 mM and 100 mM salinity stress conditions were decreased in compared to non-salinity treatment, but the growth reduction in the inoculated plants with *P. indica* was less than control plants.

**Table 2:** Analysis of variance (ANOVA) results for the main and interactive effects of *P. indica* inoculation and salinity stress on shoot and roots characteristics.

		Sum of Squares							
Source of Variation	df	Dry Mass (g)	Na/K	N (%)	Mg (mg/kg)	Ca (mg/kg)	Fe (mg/kg)	Carbohydrates (μmol/g)	Catalase (µmol/min/gr)
Shoots									
*blocks*	2	0.265	0.086	0.154	20252	408914	3.06	0.28	0.08
*P. indica*	1	5.57**	1.6**	2.12**	823900**	2777724.5**	1062.6**	74.89**	0.349**
*Salinity*	2	5**	1.99**	0.73**	659825**	2452679.38**	449.54**	86.15**	0.566**
*P. indica × Salinity*	2	0.04ns	1.04**	0.07ns	58910.4ns	33741.16ns	45.79ns	33.45**	0.047*
*Error*	10	0.08	0.06	0.05	32577.1	96785.72	33.52	2.28	0.013
*Coefficient of variation*		8.88	54.73	8.15	7.47	8.1	6.33	25.83	17.59
Roots									
*blocks*	2	0.006	0.133	0.035	1634.38	29568.5	52850	2.12	0.08
*P. indica*	1	4.38**	0.78**	1.75**	1592517.5**	1249253.5**	235069*	38.88**	0.349**
*Salinity*	2	5.69**	4.05**	0.59**	615998**	1038024.6**	200527*	39.68**	0.566**
*P. indica × Salinity*	2	0.026ns	0.41**	0.007ns	22712.05ns	103954.16ns	2925.72ns	12.97**	0.047*
*Error*	10	0.12	0.06	0.03	51972.32	54743.83	30497.58	1.14	0.013
*Coefficient of variation*		10.7	34.81	8.16	9.84	10.2	12.18	25.05	17.59

The single- and double-star asterisk indicate significant difference among treatments within individual and interactive main effects according to Fisher's LSD at 95 and 99% confidence levels, accordingly.

**Table 3:** Analysis of variance (ANOVA) results for the main and interactive effects of *P. indica* inoculation and drought stress on shoot and roots characteristics.

		Sum of Squares						
Source of Variation	df	Dry Mass (g)	N (%)	Mg (mg/kg)	Ca (mg/kg)	Fe (mg/kg)	Carbohydrates (μmol/g)	Catalase (µmol/min/gr)
Shoots								
*blocks*	3	0.01	0.01		23516.66	33.57	1.52	0.02
*P. indica*	1	0.45**	4.43**		2112266.66**	1585.67**	55.32**	1.23**
*Drought*	2	0.21**	1.55**		2869279.16**	1290.81**	79.41**	7.93*
*P. indica × Drought*	3	0.004ns	0.01ns		110454.16ns	35.18ns	14.18**	0.926**
*Error*	15	0.01	0.04		170633.4	48.08	0.83	0.098
*Coefficient of variation*		16.49	8.91	6.73	11.36	8.96	21.38	29.8
Roots								
*blocks*	3	0.06	0.03	33718	828333.3	288981	0.14	0.02
*P. indica*	1	2.37**	1.52**	1898437.5**	20535000.67**	2337504.16**	29.25**	1.23**
*Drought*	2	1.82**	0.96**	1556216.66**	14315000.67**	1092612.5**	43**	7.93*
*P. indica × Drought*	3	0.45**	0.003ns	291800.66ns	15000.0ns	37504.16ns	5.30**	0.926**
*Error*	15	0.02	0.035	98111.9	348333.3	12519.94	0.22	0.098
*Coefficient of variation*		12.9	12.99	9.05	10.49	9.1	12.88	29.8

The single- and double-star asterisk indicate significant difference among treatments within individual and interactive main effects according to Fisher's LSD at 95 and 99% confidence levels, accordingly.

A reduction of shoots and roots dry mass was observed by increasing the level of drought (**Figures 2C, D**). Increasing the level of drought stress in the control and inoculated plants caused a decrease of 40.3 and 30% of the dry mass of shoots, respectively. The highest and the lowest amount of root dry mass was observed in inoculated plants without drought stress and control plants with AWC of 25%. The root dry mass of inoculated plants with *P. indica* with 100% AWC was 1.2 times more than control plants.

### 3.2 Effect of inoculation-salinity on Na/K ratio in shoots and roots

**Figure 3:**
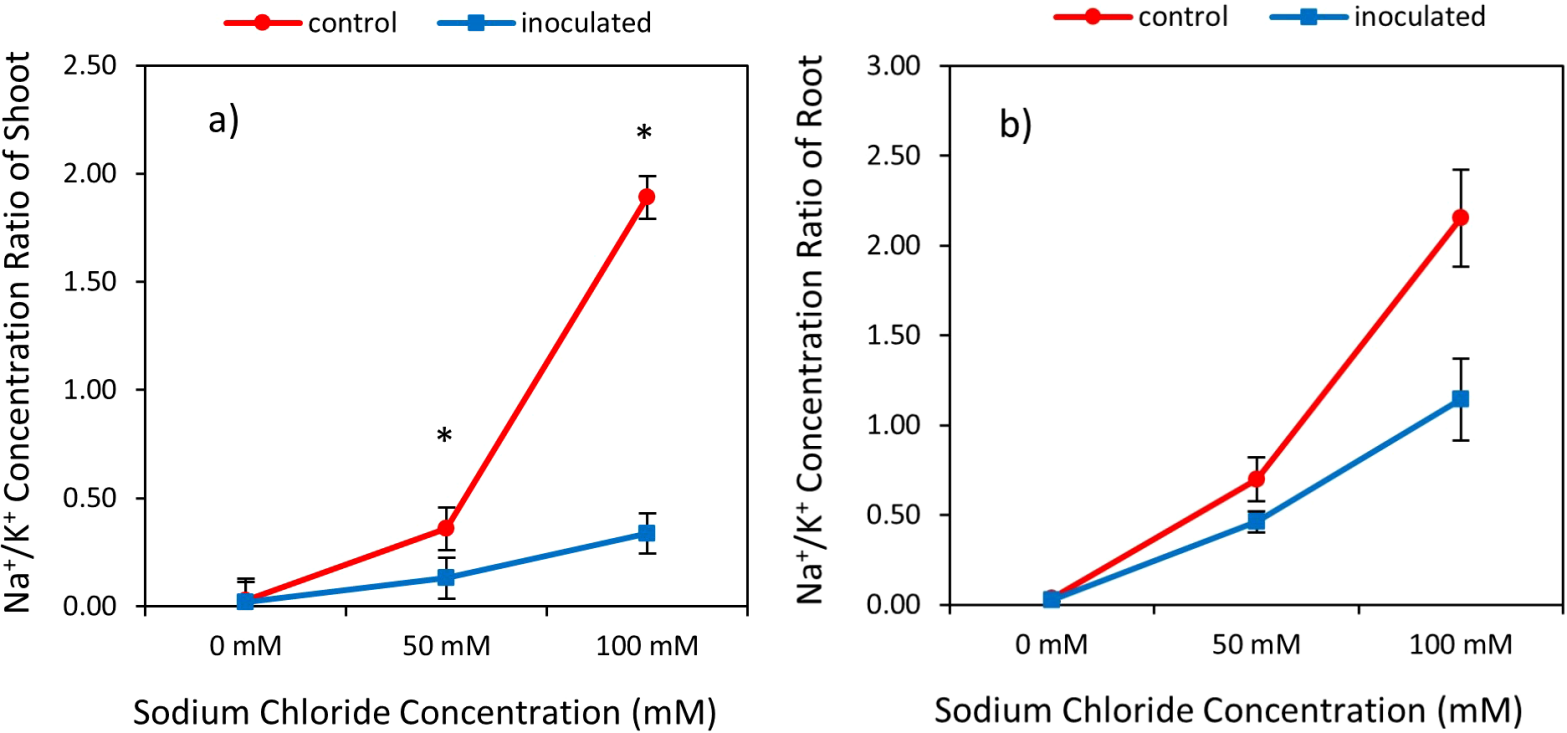
Interactive effect of *P. indica* inoculation- salinity rates on the ratio of sodium (Na) to potassium (K) Concentration in shoots **(A)** and roots **(B)**. The vertical error bars in the figures denote standard error. The single-star asterisk indicate significant difference between inoculated and control treatments at particular salinity or drought level according to paired T-test at 95% confidence level.

The result showed statistically significant effects of *P. indica*, Salinity on the ratio Na/K shoots and roots (**Table 2**). Na/K of shoots and roots was increased by raising level of salinity treatment and increasing Na/K in non-incubated plants was higher than in plants incubated with *P. indica*. Under 50- and 100-mM sodium chloride levels, Na/K in shoots of non-inoculated plants was 63.88% and 82.01% higher than in inculcated plants with *P. indica*. This ratio with increasing salinity level from 50 to 100 mM was 2.61 and 5.55 times in control and inoculated plants, respectively. In salinity level of 100 mM sodium chloride, the ratio of Na/K in control and inoculated plants showed statistically significant differences with each other and under 50- and 100-mM salinity levels, the Na/k was in control plants (non-inoculated plants) 34.28% and 46.97%, was higher than Na/K in inoculated plant under 50-, and 100-mM salinity stress. Increasing the level of salinity from 50 to 100 mM in control and inoculated plants an increase of 67.44% and 59.46% of Na/K in roots (**Figures 3A, B**).

### 3.3 Effect of inoculation-salinity and inoculation-drought on biomass N content

**Figure 4:**
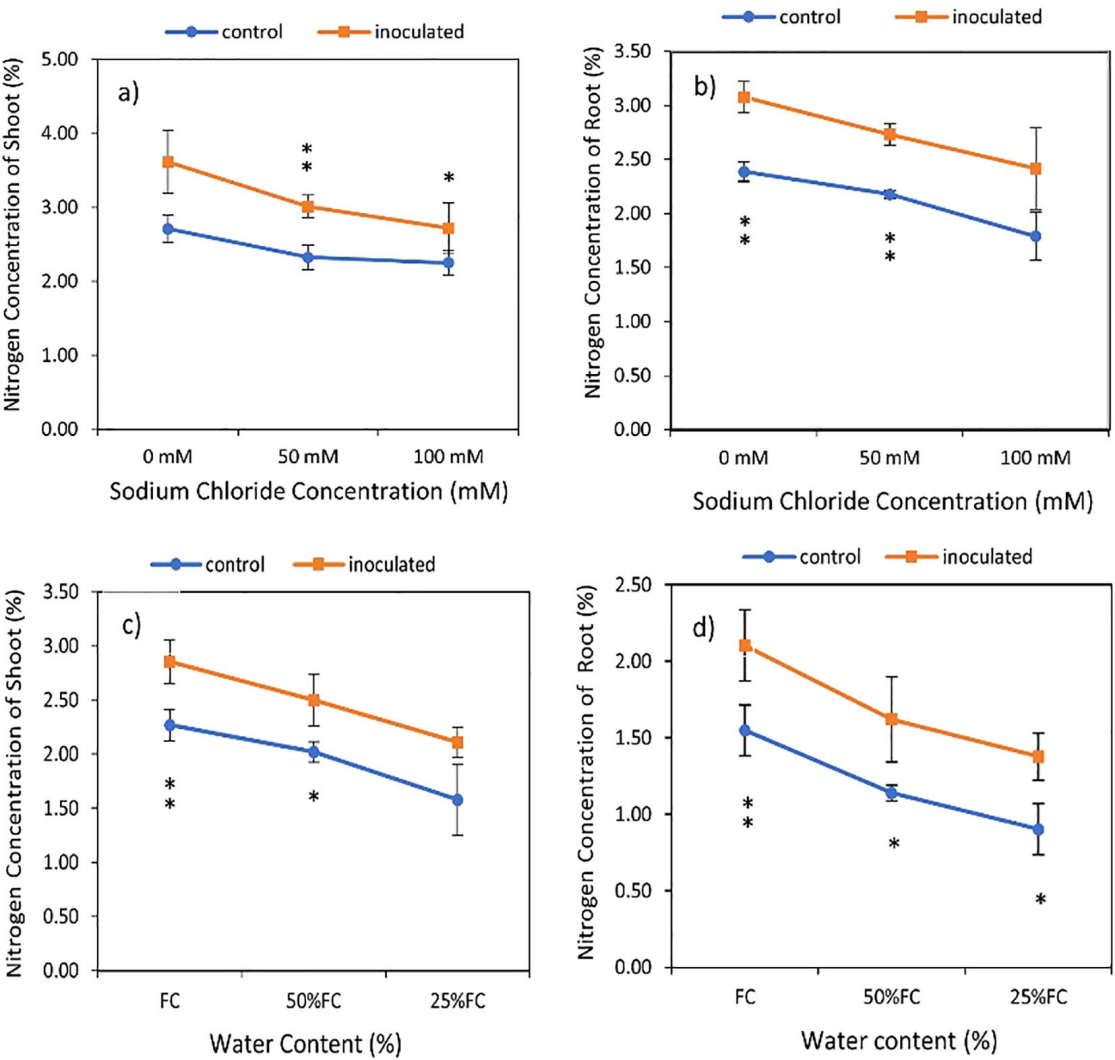
Interactive effect of *P. indica* inoculation-salinity on nitrogen (N) concentration of shoots **(A)** and roots **(B)** and the interactive effect of *P. indica* inoculation-drought on N concentration of shoots **(C)** and roots **(D)**. The single- and double-star asterisk indicate significant difference between inoculated and control treatments at particular salinity or drought level according to paired T-test at 95 and 99% confidence levels, accordingly. The vertical error bars in the figures denote standard error.

The results of the data variance analysis showed that the effect of *P.* indica × salinity (**Table 2**) and *P. indica* × drought (**Table 3**) on the shoots and roots N is significant at the level of 1%. The result indicated that the nitrogen concentration of shoots and roots with *P. indica* inoculation has a significant difference in comparison to those without *P. indica* inoculation under salinity treatment. The highest amount of shoots nitrogen belongs to inoculated plants under optimal conditions in terms of salinity and the lowest one is related to control plants at the level of 100 mM (**Figures 4A, B**). Increasing the salinity level caused a decrease in the nitrogen percent of shoots and roots in both inoculated and non-inoculated plants. The amount of nitrogen in plants inoculated with *P. indica* showed a significant correlation with each other at all levels of salinity stress. However, the decrease in nitrogen percentage of control plants was not significant in 50 and 100mM salinity stresses (**Figures 4A, B**).

Based on **Table 3**, the nitrogen concentration of shoots and roots under inoculated plants had a significant difference with control plants (non-inoculated plants) in drought treatments, although the increase in drought stress caused a decrease in the nitrogen concentration of the shoots and roots in the control and inoculated plants (**Figures 4C, D**). The highest and the lowest nitrogen percentage were observed in inoculated plants grown in no drought stress and control plant (noninoculated) in 25% available water (**Table 2**). Under 100% available water, the nitrogen percentage of shoots was 1.2 times higher than the nitrogen percent of plants inoculated with *P. indica*. Also, increasing drought stress levels from 50% FC to 25% in the control and inoculated plants caused a decrease of 18.06 and 24.87 percent of nitrogen concentration, respectively (**Figure 4C**). The difference in percentage of nitrogen in the roots of plants inoculated with *P. indica* at the levels of 100% AWC, 50% AWC, and 25% AWC with the control plants was statistically significant (at the level of 5%) (**Figure 4D**).

### 3.4 Effect of inoculation- salinity and inoculation- drought on biomass Mg content

**Figure 5:**
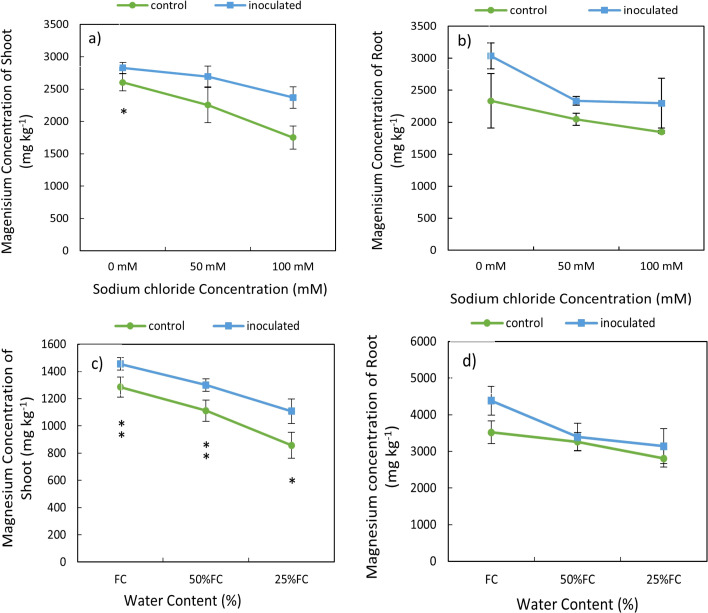
Interactive effect of *P. indica* inoculation-salinity on magnesium (Mg) Concentration of shoots **(A)** and roots **(B)** and the interactive effect of *P. indica* inoculation-drought on Mg of shoots **(C)** and roots **(D)**. The single- and double-star asterisk indicate significant difference between inoculated and control treatments at particular salinity or drought level according to paired T-test at 95 and 99% confidence levels, accordingly. The vertical error bars in the figures denote standard error.

The results of variance analysis indicated that the effect of *P. indica* × salinity (**Table 2**) and *P. indica* × drought (**Table 3**) on Mg of shoots and roots was significant at 1% level. Increasing the level of salinity stress reduced the Mg concentration in shoots and roots. The Mg concentration in the aerial parts of the control plants showed a significant difference in all 3 different salinity levels, but this difference in the inoculated plants was observed only in 50- and 100-mM salinity levels (**Table 2**). The reduction of Mg concentration in shoots of non-inoculated plants (control plants) under 50- and 100-mM salinity stress was 13.48% and 33%, respectively. However, in the plants inoculated with *P. indica*, shoots Mg concentration was reduced by 12% more than moderate salinity stress (**Figures 5A, B**). The decrease in root Mg concentration in control plants compared to plants inoculated with *P. indica* was significant at three salinity levels of 0, 50 and 100 mM, and the amount of this reduction was 23.1, 12.34, and 19.53%, respectively (**Figure 5D**).

Increasing drought also caused a decrease in the magnesium concentration of shoots and roots in inoculated and non-inoculated treatments (**Table 3**). The reduction of Mg concentration in shoots and roots in control plant (non-inoculated) was higher than in inoculated plant with *P. indiaca* under different levels of drought stresses. The difference in Mg concentration of shoots and roots of plants inoculated with *P. indica* in different levels of available water including 100%, 50%, and 25% AWC with control plants were statistically significant (at the 5% level) (**Figures 5C, D**).

### 3.5 Effect of inoculation-salinity and inoculation-drought on biomass Ca content

**Figure 6:**
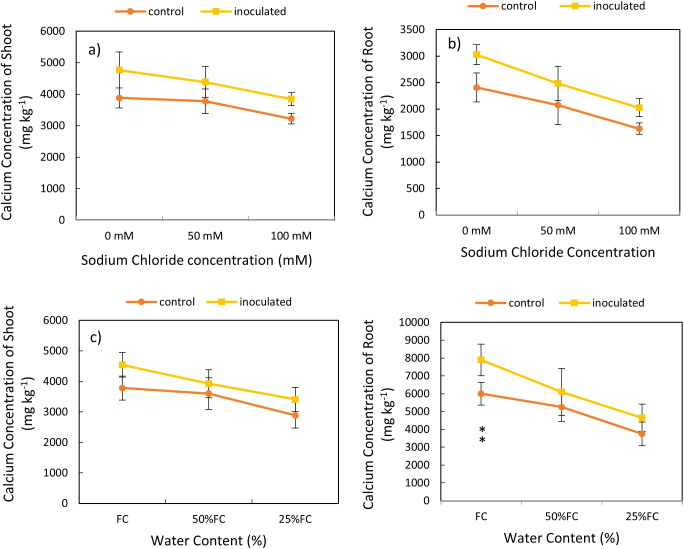
Interactive effect of *P. indica* inoculation-salinity on calcium (Ca) Concentration of shoots **(A)** and roots **(B)** and the interactive effect of *P. indica* inoculation-drought on Ca concentration of shoots **(C)** and roots **(D)**. The single- and double-star asterisk indicate significant difference between inoculated and control treatments at particular salinity or drought level according to paired T-test at 95 and 99% confidence levels, accordingly. The vertical error bars in the figures denote standard error.

Based on the variance analysis, we found that that the effect of *P. indica* × salinity (**Table 2**) and *P. indica* × drought (**Table 3**) on shoots and roots Ca concentration was significant at 1% level. The mean comparison showed that the increase in salinity level caused a decrease in the Ca concentration in shoots and roots. The difference between Ca concentration of the shoots and roots in 50-, and 100-mM salinity levels was significant statistically (**Figures 6A, B**). The Ca concentration in the control plants showed a significant decrease when the salinity stress increased significantly, and this reduction was not significant in 50 mM sodium chloride salinity level. The amount of calcium in the roots of plants inoculated with *P. indica* was significantly higher than the control samples at all salinity levels. The root Ca concentration of the plants inoculated with *P. indica* at 0, 50 and 100 levels, 20.48, 12.4-, and 18.38-mM sodium chloride (salinity stress) were reported higher than the control samples, respectively (**Figures 6A, B**).

The mean comparison demonstrated that the increase in drought level caused a reduction in the Ca concentration in shoots and roots (**Figures 6C, D**). The highest amount of calcium concentration in the shoots was observed in plants with *P. indica* inoculation and grown under no drought stress and the lowest amount of calcium concentration in the shoots belongs to the control plant under 25% available water (**Table 3**). The results showed that in optimal conditions in terms of drought stress (100% AWC), the calcium concentration of the shoots inoculated with the *P. indica* was about 1.2 times that of the control plants. The concentration of calcium in the shoots of control plants and plants inoculated with the *P. indica* under 25% AWC were statistically significantly different from each other, so that the Ca concentration in plants inoculated with *P. indica* at 25% AWC level was 22.66% more than the control plants. The difference in the concentration of calcium in the roots of plants inoculated with *P. indica* at the levels of 100%, 50% and 25% AWC with the control plants was statistically significant (at the 5% level) (**Figure 6D**).

### 3.6 Effect of inoculation-salinity and inoculation-drought on biomass Fe content

**Figure 7:**
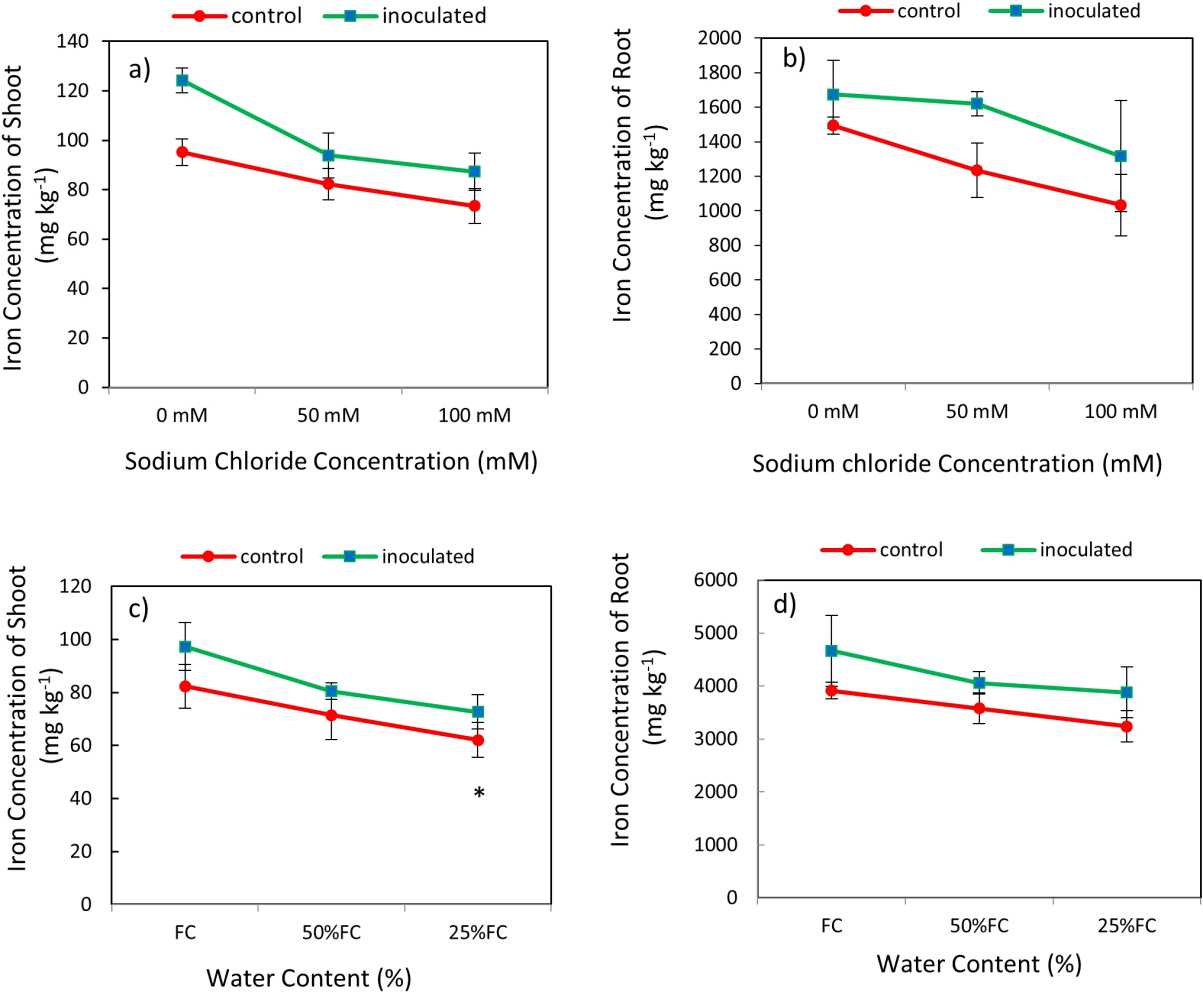
Interactive effect of *P. indica* inoculation-salinity on iron (Fe) Concentration of shoots **(A)** and roots **(B)** and the interactive effect of *P. indica* inoculation-drought on Fe concentration of shoots **(C)** and roots **(D)**. The single- and double-star asterisk indicate significant difference between inoculated and control treatments at particular salinity or drought level according to paired T-test at 95 and 99% confidence levels, accordingly. The vertical error bars in the figures denote standard error. Increasing salinity stress from 0 to 100 mM sodium chloride did not cause any significant difference in the Fe concentration of roots.

The result showed that the effect of *P. indica* × salinity (**Table 2**) and *P. indica* × drought (**Table 3**) on the Fe concentration of the shoots and roots at 1% was significant statistically. Increasing the salinity level decreased Fe concentration in inoculated and non-inoculated (control) plants. The Fe concentration of plant shoots inoculated with *P. indica* at different salinity levels have significant differences with each other, but in the case of control plants, this difference was not significant between 50- and 100-mM salinity levels. Fe concentration of the plant shoots inoculated with *P. indica* was significantly higher than control plants at 50- and 100-mM sodium chloride salinity levels. The Fe concentration of the plant shoots inoculated with *P. indica* at the levels of 50- and 100-mM sodium chloride, respectively, was reported to be 18.91 and 19.64 higher than Fe concentration in control plants (**Figure 7A**). Increasing salinity stress from 0 to 100 mM sodium chloride did not cause any significant difference in the Fe concentration of roots (**Figure 7B**). Increasing salinity stress causes a decrease in root iron concentration in both groups of control plants and plants with *P. indica* and the reduction of Fe concentration in the control plants under 50- and 100-mM sodium chloride was 23.88 and 21.51% more than the plants inoculated with *P. indica* (**Figure 7D**).

The highest amount of iron concentration was observed in plants with *P. indica* inoculation and grown under no drought stress and the lowest amount of iron concentration belongs to the control plants (no *P. indica* inoculation) under 25% AWC (**Table 2**). The shoot Fe concentration in control plants and plants inoculated with *P. indica* under 25% and 50% AWC have statistically significant differences with each other, so shoot Fe concentration in plants inoculated with the *P. indica* was 24.15% and 27.61% higher than the control plants at 50% AWC and 25% AWC levels, respectively. Increasing the drought stress level from FC to 50% and 25% of FC and in the control and inoculated plants caused a decrease of 9.69 and 13.24% in the Fe concentration of aerial parts, respectively (**Figure 7C**). The results of the mean comparison showed that the increase in drought stress caused a decrease in root iron concentration in the studied plants.

The highest amount of iron in shoot and root related to the plants with *P. indica* inoculation and grown in the condition of no drought stress and the lowest amount of shoot and root iron belongs to the control plants (no *P. indica* inoculation) under 25% AWC (**Table 3**). The root iron concentrations of the control plants and inoculated plants with *P. indica* under 25% and 50% AWC have statistically significant differences with each other. So, root iron concentration in plants inoculated with *P. indica* at 50% and 25% AWC were 11.83% and 16.5% more than the control plants, respectively (**Figure 7D**).

### 3.7 Effect of inoculation-salinity and inoculation-drought on carbohydrates

**Figure 8:**
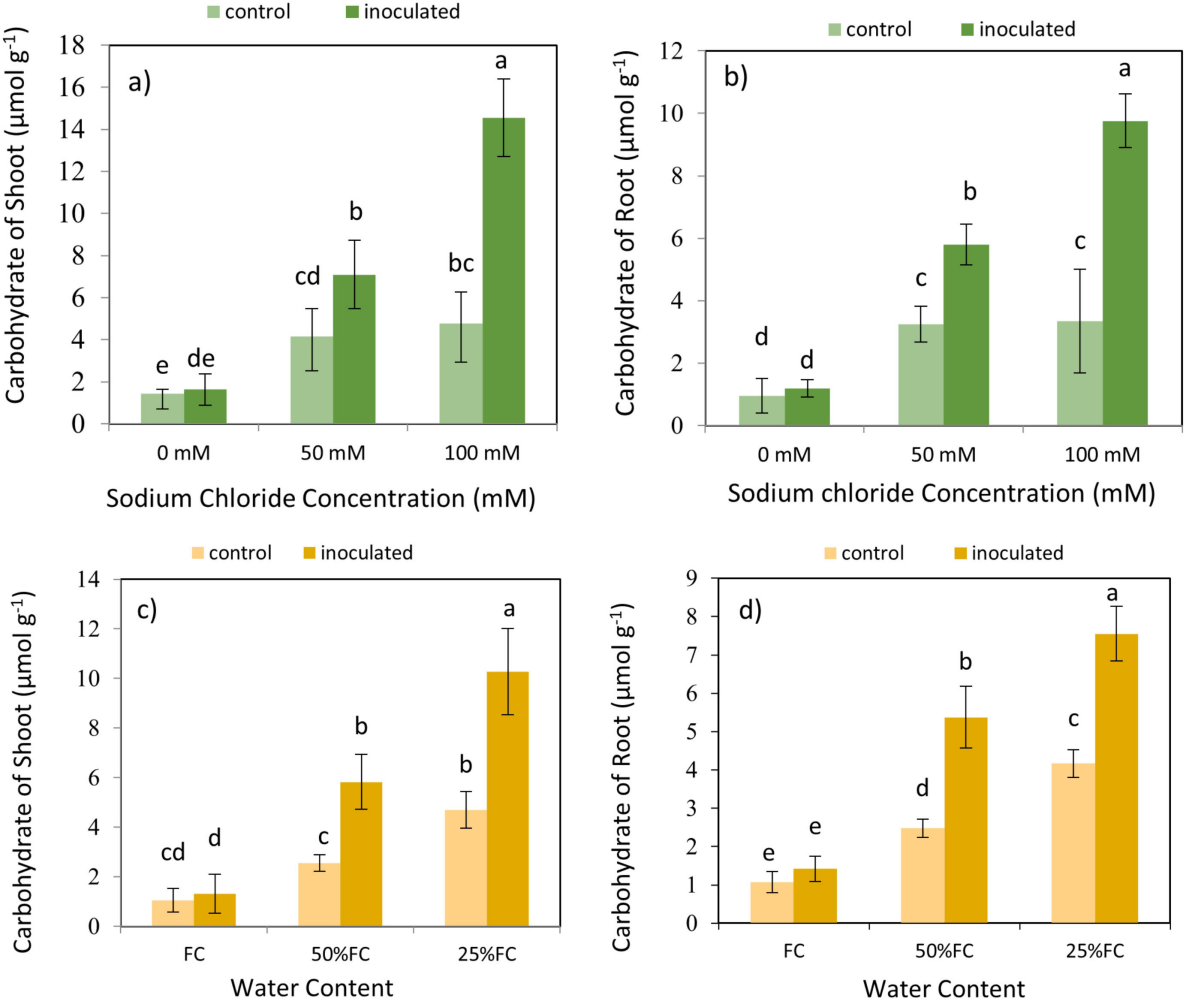
Interactive effect of *P. indica* inoculation and salinity on carbohydrate concentration in shoots **(A)** and roots **(B)**, and interactive effect of *P. indica* inoculation and drought on carbohydrate concentration in shoots **(C)** and roots **(D)**. Letter annotations indicate significant differences among treatments resulting from interactions between inoculation-salinity, and inoculation-drought, according to Fisher’s LSD test (p < 0.05). Vertical error bars represent standard errors.

The result expressed that the effect of *P. indica* × salinity (**Table 2**) and *P. indica* × drought (**Table 3**) on the carbohydrates of shoots and roots is statistically significant at 1% level. By increasing salinity stress, the carbohydrates concentration in shoots and roots of the plants inoculated with the *P. indica* and control plants (without inoculation with *P. indica*) increased. The results showed that in plant shoot and root inoculated with p. indicia, the optimal level of salinity (without salinity stress) had a significant difference with 50- and 100-mM sodium chloride of salinity stress, and the difference between the salinity stress of 50- and 100-mM sodium chloride was also significant statistically. In control plants (without inoculation), the increase in salinity stress at all levels did not cause significant differences in the concentration of carbohydrates in the roots (**Figures 8A, B**).

The increase in drought stress caused an increase in the concentration of carbohydrates in the shoots and roots of the plants inoculated with *P. indica* and control plants (**Figures 8C, D**). In shoots and roots of plants inoculated with *P. indica*, the optimal moisture stress (100% AWC) has a significant difference with the 50% and 25% AWC. In control plant (without inoculation) under optimal moisture stress (100% AWC) had a significant difference with the extreme dryness level (25% AWC) however, this difference was not significant with the medium moisture level (50% AWC). In contrast, the reduction of carbohydrates concentration under 3 different drought stresses was significant in control plants (**Figures 8C, D**). Increasing drought stress increased the concentration of carbohydrates in plants inoculated with P-India and control plants (without inoculation).

### 3.8 Effect of inoculation-salinity and inoculation-drought on catalase enzyme of shoots and roots

**Figure 9:**
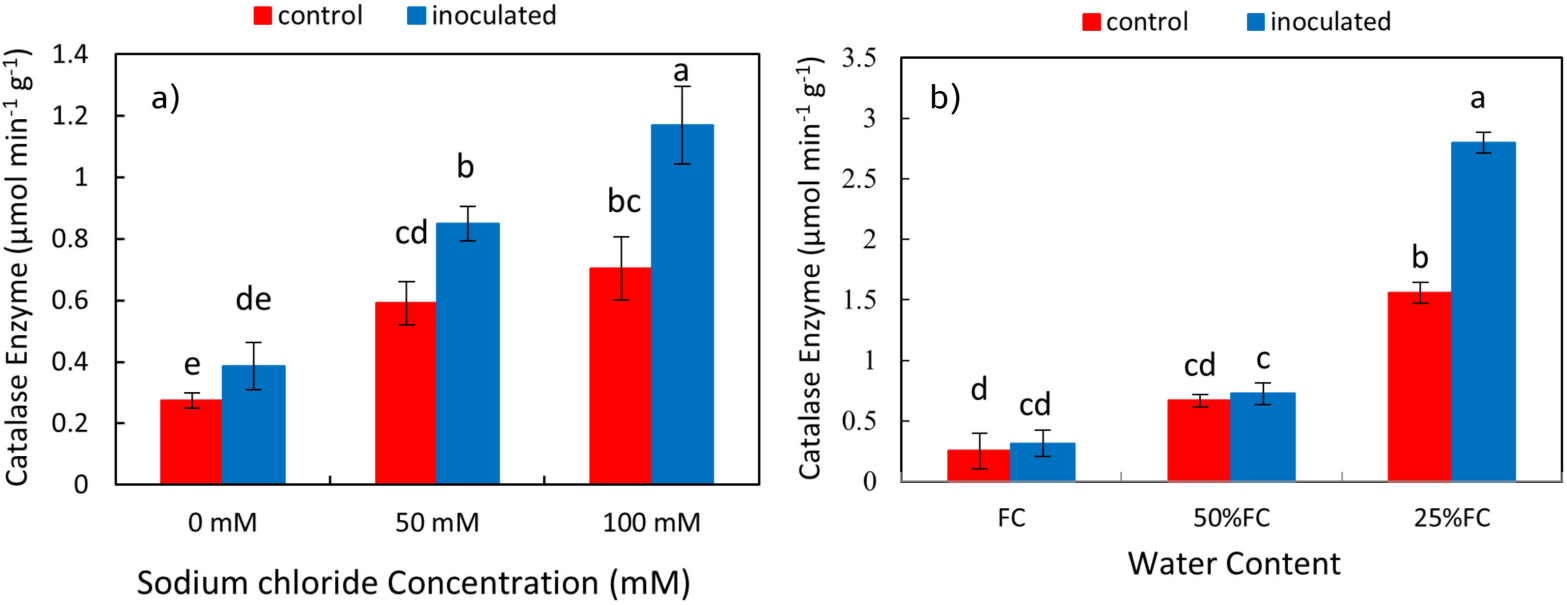
Interactive effect of *P. indica* inoculation on catalase enzyme activity in shoots under salinity **(A)** and drought **(B)** conditions. The letter annotations indicate significant differences among treatments resulting from the interaction between inoculation, salinity, and drought, according to Fisher’s LSD test (p < 0.05). Vertical error bars represent standard errors.

The effect of *P. indica* × salinity (**Table 2**) and *P. indica* × drought (**Table 3**) on the concentration of Catalase enzyme of the shoots and roots was significant at 1% level. The results of the mean comparison indicated with the increase of salinity stress, the concentration of catalase in the plant shoots has increased. In the plant inoculated with the *P. indica*, increasing salinity stress at all levels significantly increased the catalase enzyme concentration of the shoots. In control plants (without inoculation), the optimal level of salinity (without salinity) had a significant difference with the levels of 50- and 100-mM sodium chloride, but the difference between the levels of 50- and 100-mM sodium chloride in the control plants was not significant. Increasing salinity stress in control and inoculated plants with *P. indica* increased the concentration of catalase enzyme, and this increase in the plants inoculated with the *P. indica* at the salinity levels of 50 and 100 mM of sodium chloride was 29.76 and 39.65% of the control plants, respectively (**Figure 9A**). The increase in drought stress caused an increase in the concentration of catalase enzyme in the shoots of the rice. The result showed that in plants inoculated with *P. indica*, the optimal dryness level (FC) had no significant difference with 50% FC, but the difference with 25% FC was significant. Also, the difference between the concentration of Catalase enzyme in the drought levels with 50% FC and 25% FC in non-inoculated plants was significant. The concentration of catalase enzyme in shoots of the plant inoculated with *P. indica* under 50% FC and 25% FC was 10 and 42.59% higher than the concentration of catalase enzyme in the control plant (**Figure 9B**).

### 3.9 Hierarchical clustering

**Figure 10:**
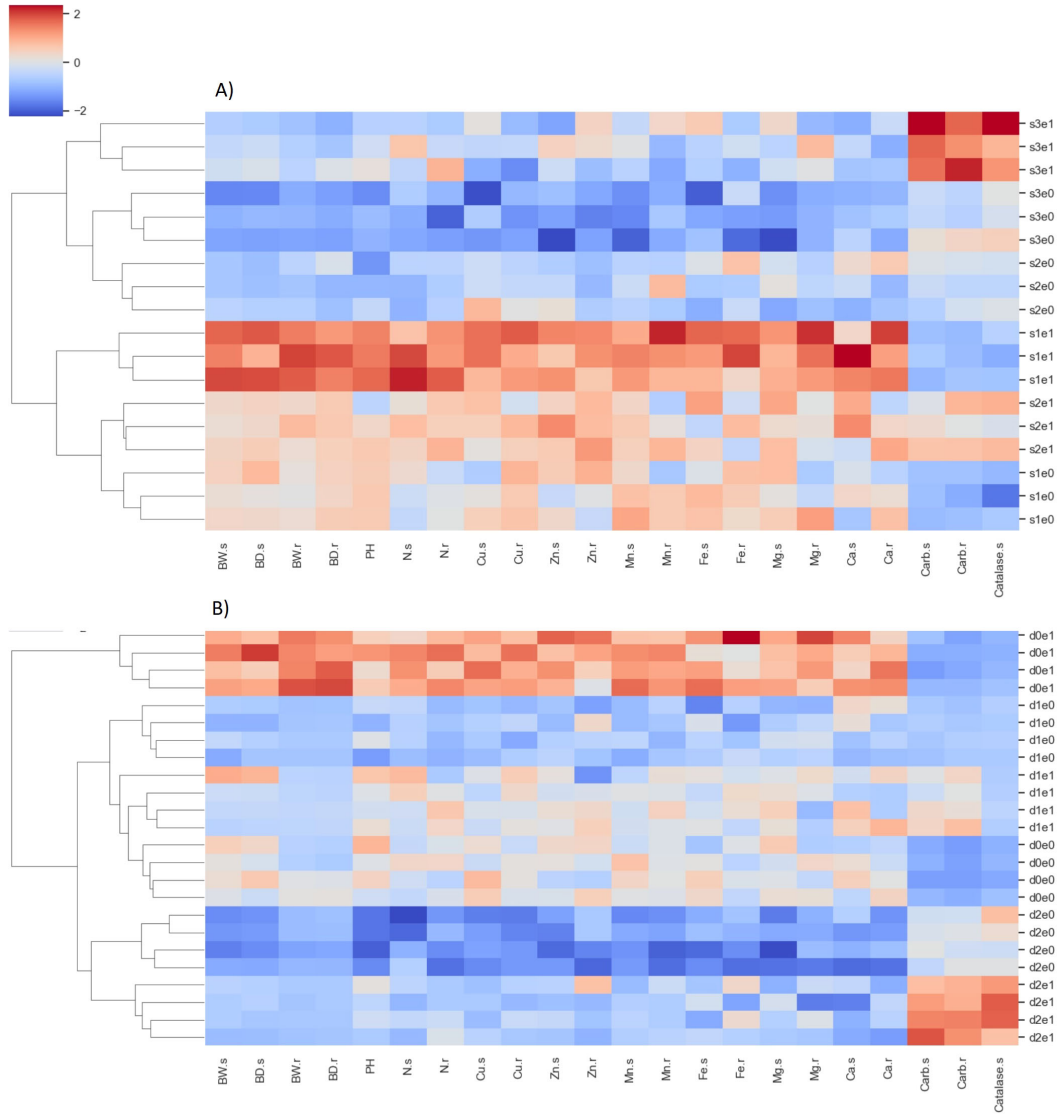
Heatmap illustrating the results of hierarchical clustering analysis based on Euclidean distance and Ward’s functions. Each cell displays a standardized value corresponding to nutrient content and biomass in plants subjected to salinity **(A)** and drought **(B)** stresses. The analysis includes treatments with all four replicates. Plant properties labels on the x-axis are suffixed with s and r, denoting shoots and roots, respectively. Treatment labels on the y-axis, such as s1-3 and d0-2, indicate the level of salinity and drought, respectively. Abbreviations e0 and e1 denote the absence or presence of *P. indica* inoculation.BW is weight biomass, BD is dry biomass, PH is plant height.

Clustering analysis yielded distinct differentiation among treatments under exposures to both salinity and drought stress (**Figure 10**). Dendrogram analysis revealed that, under salinity stress, the initial level of separation occurred between treatments S2E0, S3E0, and S3E1 on one branch, and treatments S1E1, S2E1, and S1E0 on another. Here, S1-3 denotes salinity levels ranging from 0 to 100 mM, and E0-1 indicates the absence and presence of inoculation, respectively. The former group exhibited elevated catalase enzyme and carbohydrate levels, alongside reduced root and shoot mass and nutrient content, while the latter group demonstrated the opposite trend. The only exception occurred in S2E1 where despite the relatively high nutrient content and biomass, also had relatively high catalase and carbohydrate content in roots and shoots. The secondary level of clustering grouped replicates of each treatment. Under drought stress, the primary level of grouping separated the treatments D0E1 with all other treatments. Here D0-2 stands for drought intensities 0 to 25% FC, respectively. As expected, D0E1, resulted in the lowest rate of catalase enzyme and carbohydrate accumulation in root and shoot biomass and highest biomass and nutrient content.

## 4 Discussion

The elevated salinity levels led to a reduction in both root and shoot biomass dry weight, irrespective of whether the plants were inoculated or non-inoculated. This anticipated response can be attributed to the reduced osmotic potential and ion toxicity, which adversely affect photosynthetic efficiency and overall plant vigor (Pourraeisi et al., 2022). The adverse effect of increasing salinity was evident in the reduction of leaf area (**Figure 1**). However, seed inoculation with *P. indica* significantly enhanced crop tolerance to escalating salinity levels, thus preserving biomass. Specifically, rice seeds that were inoculated led to nearly identical or greater root and shoot biomass at 100 mM of salinity compared to non-inoculated plants at 50 mM The enhanced resilience to salinity resulting from *P. indica* inoculation has been attributed to increases in proline and antioxidants, along with decreased concentrations of malondialdehyde (Nassimi and Taheri, 2017; Saddique et al., 2018; Reshna et al., 2022). Proline accumulation naturally transpires in the cytoplasm, where it functions as an osmoprotectant, safeguarding against osmotic stress. A high cytosolic proline level lowers cellular water potential compared with the external water potential, facilitating water influx into the cells to uphold cell turgidity (Nguyen et al., 2021). The accumulation of proline induces oxidative stress by eliciting antioxidant enzymes to counteract malondialdehyde, indicative of membrane malfunctioning (Khan et al., 2022). Likewise, drought stress led to a significant reduction of root and shoot biomass. Significant interaction between drought intensity and inoculation indicates a greater effect of drought on reduction of root than shoot biomass. This is due to the plant's natural response to water stress. When faced with drought conditions, rice plants prioritize water conservation and shoot growth over root development to maintain hydration and photosynthetic activity (Wassmann et al., 2009; Hassan et al., 2023). Drought stress reduced the leaf area and plant dry weight (Choudhury et al., 2022). The adverse impact of drought on leaf development is likely due to water deficit's influence on cell size and meristematic cell division. It appears that drought perturbs the development of primary leaf cells and their differentiation, resulting in a reduction in both leaf number and area (Lobato et al., 2008). While a reduction in plant biomass was observed in both inoculated and non-inoculated plants, the rate of reduction was significantly higher in noninoculated plants. Seed inoculation with *P. indica* enhanced drought tolerance, resulting in similar biomass compared to non-inoculated plants with 25% less water content. *P. indica*-inoculated plants have demonstrated improved photosynthetic efficiency (Tsai et al., 2020), attributed to the *P. indica*’s effect on enhancing the antioxidative capacity of plants. This enhancement is evident in the reduction of lipid peroxidation induced by reactive oxygen species (Zhang et al., 2021). Therefore, the observed significant increase in catalase enzyme activity as an antioxidant in P. indica-inoculated plants is directly associated with the greater root and shoot biomass.

An increase in Na to K ionic concentration within plant shoots and roots is a typical characteristic of salt-affected plants (Zhu, 2016). One defense mechanism enhancing plant tolerance to salinity involves the maintenance of a high K/Na in plant organs. Although increased salinity levels increased the Na/K ratio in the shoots and roots of all rice plants, we observed a lower Na/K ratio in the shoot and root tissue of plants colonized with *P. indica* under salt stress compared to non-inoculated plants. The Na/K ratio exceeded 7-fold in non-inoculated plant shoots compared to inoculated plants’ shoots. The higher tolerance of *P. indica*-inoculated plants to Na accumulation can be attributed to enhanced transcript levels of genes encoding the high-affinity Potassium Transporter and inward-rectifying K channels, pivotal in regulating Na and K homeostasis (Abdelaziz et al., 2017).

The enhanced tolerance observed in *P. indica* inoculated plants corresponded to increased uptake of N, Mg, Ca, and Fe by plants, as evidenced by higher content in both root and shoot biomass than the control group. Similar observations have been reported in literature (Verma et al., 2022). Drought and salinity share similar mechanisms in diminishing nutrient transport to plants, as both are rooted in reduced water transport. The mechanisms governing nutrient absorption and transfer in plants, including mass flow, diffusion, and root interception, are all contingent upon the soil moisture content. A decrease in soil moisture and/or rise in salinity levels leads to a reduction in solute transport (Zhang et al., 2022), as demonstrated in the literature across various nutrients (Cosme et al., 2016). When the exchange capacity of the soil becomes saturated with sodium beyond 40 to 50%, it can lead to nutritional disorders and disturb the cation balance in the soil solution. Similarly, drought diminishes the rate of nutrient diffusion from the soil environment to the root absorption surface, coinciding with a decrease in soil moisture (Rezapour et al., 2023). The accumulation of Cl^-^ and a reduction in the concentration of essential elements such as K and Mg in the roots and leaves of certain species, including grasses, alfalfa, and rice, have also been documented under drought stress (Choudhury et al., 2022). The facilitated uptake of essential nutrients with *P. indica* inoculation is attributed to multiple mechanisms. Changes in root architecture, such as increased root length, surface area, and branching, have been shown to facilitate greater soil exploration for nutrients, particularly under stress conditions. Research has demonstrated that *P. indica*-inoculated rice plants exhibit enhanced root growth and morphology compared to non-inoculated plants, especially under drought and salinity stresses (Sharma et al., 2023).

Additionally, *P. indica* colonization triggers various biochemical and physiological changes in plants, including the upregulation of genes involved in nutrient assimilation and metabolism pathways (Yadav et al., 2010; Jia et al., 2012)

Inoculation with *P. indica* resulted in an increase in Ca content in both roots and shoots. Under salinity conditions, Ca plays a pivotal role in regulating ion transfer to plant cells. This ion induces various physiological reactions and influences membrane structure and ion displacement. Consequently, by elevating the concentration of Ca around the roots, its accumulation in both leaves and roots increases, subsequently leading to an increase in Kion levels while decreasing Cl^-^ and Na concentrations. Additionally, Ca hampers the translocation of Na from roots to leaves, thus enhancing tolerance to salt stress (Rezapour et al., 2022). The reduction in soil moisture diminishes the transport of Ca from the soil to the root surface, and plant Ca absorption becomes dependent on transpiration rates. Consequently, under drought stress, a decrease in transpiration rate leads to a reduction in calcium absorption (Borna et al., 2021). Bartels and Sunkar (2005) noted that the decrease in Ca content in wheat plants is attributed to reduced Ca absorption rates due to water deficiency and the absence of necessary conditions *f*or root absorption.

The mobility of nutrients in soil varies significantly depending on various factors. While root interception plays a major role in nutrient uptake when nutrients are readily available in the solution adjacent to rootlets or mycorrhizal hyphae, mass flux and diffusion become predominant mechanisms for transporting nutrients from distant locations to the root area. Fe along with P and K is among the low-mobility nutrients whose transport relies primarily on diffusion, driven by energy gradients (Havlin et al., 1999). This mechanism is crucial for nutrient uptake, especially considering that drought and salinity conditions typically restrict root extension and the potential for direct interception (Zhang et al., 2021). Our observations indicate that *P. indica* inoculation increased Fe uptake under both salinity and drought conditions, consistent with findings from previous studies. This enhancement corresponds to the inoculation’s impact on maintaining root biomass, which, in turn, facilitates water and nutrient scavenging by roots, thereby sustaining Fe diffusion under environmental stresses.

Accumulation of carbohydrates such as sugars and starches are a plant defense mechanism against environmental stresses. Therefore, quantifying carbohydrates is an effective approach to assessing stress levels. This response serves several purposes, including energy storage, osmotic adjustment, and protection against oxidative damage (Krasensky and Jonak, 2012; Keunen et al., 2013; Singh et al., 2022). Our findings align closely with previous observations indicating increased carbohydrate accumulation in both plant shoots and roots under escalating salinity and drought conditions (Dadkhah, 2010). The findings from Khalid (2006) study on two species, *Ocimum basilicum* and *Ocimum americanum*, revealed a positive correlation between soil moisture stress and the concentration of soluble sugars. Greater accumulation of carbohydrates was found in the roots compared to the shoots of rice plants. This result can be attributed to the roots’ role as the primary organs sensing and responding to stress signals in the soil environment. Furthermore, carbohydrates stored in roots serve as reserves for subsequent regrowth and recovery post-stress alleviation (Bowne et al., 2012)

Catalase enzyme activity is an important indicator of both salinity and drought stresses. We observed an increase in catalase enzyme activity under increasing levels of salinity and drought stresses. This rise exhibited a linear trend in response to increasing salinity levels, while demonstrating an exponential trend with enhanced drought intensity. Under drought and salinity stresses, plants often undergo high production of reactive oxygen species (ROS) due to disrupted cellular processes and the accumulation of toxic compounds. ROS, particularly hydrogen peroxide (H_2_O_2_), can induce cellular damage by oxidizing macromolecules such as proteins, lipids, and nucleic acids. By enhancing the activity of antioxidant enzymes like catalase, plants can mitigate the oxidative effects of H_2_O_2_ on the Calvin cycle within chloroplasts (Shen et al., 1997). Wang et al. (2009) have noted that both drought and salinity stresses can lead to an increase in catalase enzyme activity. Their findings suggest that by enhancing antioxidant enzymes such as catalase, plants may exhibit improved tolerance to drought and salinity stress, particularly during the germination stage.

Hierarchical cluster analysis revealed that inoculated plants exposed to 100 mM salinity level exhibited the highest carbohydrate accumulation in both roots and shoots, along with the highest catalase enzyme activity. This finding underscores the substantial potential of *P. indica* inoculation in increasing crop resilience under abiotic stresses through increasing carbohydrate accumulation in plant tissues. It is widely recognized that carbohydrates, particularly soluble sugars such as sucrose, glucose, and fructose, serve as osmoprotectants by maintaining cellular turgor and osmotic balance under stress conditions (Lemoine et al., 2013). Also, carbohydrates are the primary source of energy for metabolic processes in plants. Under abiotic stress, plants often experience metabolic alterations and increased energy demand to sustain stress responses and repair cellular damage. Therefore, the increased accumulation of carbohydrates enables plants to meet the increased energy requirements for stress adaptation and recovery. The highest concentration of nutrients and plant biomass was associated with 0- and 50-mM salinity levels with inoculation. Conversely, treatments with 50- and 100-mM salinity levels without inoculation yielded the lowest biomass nutrient content and plant biomass. A similar association of physiological and metabolic properties was observed in treatments subjected to drought stress, indicating the similarity in the effects of drought and salinity on plant physicochemical properties.

## 5 Conclusion

The experiment demonstrated a reduction in the dry mass of both shoots and roots with increasing salinity levels in plants, whether inoculated or non-inoculated with *P. indica*. However, inoculation of plant shoots with *P. indica* resulted in increased dry biomass in both shoots and roots under salinity stress, highlighting the potential benefits of *P. indica* for enhancing plant growth and resilience in saline conditions. Moreover, *P. indica* inoculation exhibited positive effects on plant properties not only under stress but also under normal conditions. Increasing salinity levels led to reductions in nitrogen, magnesium, and calcium concentrations in both shoots and roots. Catalase enzyme activity, as an antioxidant enzyme, increased with rising salinity stress levels in both inoculated and non-inoculated plants. Carbohydrate concentration also increased with increasing salinity levels in the shoots and roots of both inoculated and non-inoculated plants. Inoculation of plants with *P. indica* significantly elevated carbohydrate concentrations at various sodium chloride salinity levels compared to control plants, emphasizing the positive impact of *P. indica* on carbohydrate accumulation under saline conditions. Drought stress resulted in a significant reduction in the dry mass of shoots and roots in both inoculated and non-inoculated plants. Additionally, drought significantly decreased the absorption rate of nitrogen, calcium, and magnesium by plant roots. However, this reduction in nutrient absorption by roots was less pronounced in inoculated plants with *P. indica* compared to control plants. Furthermore, the concentration of carbohydrates increased in both inoculated and non-inoculated plants with increasing drought levels, with a significantly higher increase observed in plants inoculated with *P. indica*. Catalase enzyme activity also increased with rising drought stress levels. These findings underscore the potential of *P. indica* inoculation in mitigating the adverse effects of salinity and drought stresses on rice plant growth and nutrient uptake. The enhanced plant resilience observed in this study has broader implications for improving agroecosystem resilience in the face of increasing climatic stresses and terrestrial deteriorations. By promoting plant growth, nutrient uptake, and stress tolerance, *P. indica* inoculation has the potential to contribute to sustainable agriculture practices and enhance crop productivity in challenging environmental conditions.

## Data Availability

The raw data supporting the conclusions of this article will be made available by the authors, without undue reservation.
